# Effect of a Universal Postpartum Nurse Home Visiting Program on Child Maltreatment and Emergency Medical Care at 5 Years of Age: A Randomized Clinical Trial

**DOI:** 10.1001/jamanetworkopen.2021.16024

**Published:** 2021-07-07

**Authors:** W. Benjamin Goodman, Kenneth A. Dodge, Yu Bai, Robert A. Murphy, Karen O’Donnell

**Affiliations:** 1 Sanford School of Public Policy, Duke University, Durham, North Carolina; 2 Department of Psychiatry and Behavioral Sciences, Duke University School of Medicine, Durham, North Carolina

## Abstract

**Importance:**

The Family Connects (FC) program, a community-wide nurse home visiting program for newborns, has been shown to provide benefits for children and families through the first 2 years of life. Potential longer-term outcomes for child well-being remain unknown.

**Objective:**

To determine the effect of randomization to FC on child maltreatment investigations and emergency medical care through 5 years of age.

**Design, Setting, and Participants:**

In this randomized clinical trial, families of all 4777 resident births in Durham County, North Carolina, from July 1, 2009, to December 31, 2010, were randomly assigned to receive the FC program or treatment as usual. Impact evaluation was on an intent-to-treat basis and focused on a subsample of 549 families randomly selected from the full population and included review of hospital and Child Protective Services (CPS) administrative records. Statistical analysis was conducted from November 6, 2020, to April 25, 2021.

**Interventions:**

The FC programs includes 1 to 3 nurse home visits beginning at the infant age of 3 weeks designed to identify family-specific needs, deliver education and intervention, and connect families with community resources matched to their needs. Ongoing program engagement with service professionals and an electronic resource directory facilitate effective family connections to the community.

**Main Outcomes and Measures:**

Two primary trial outcomes were CPS-recorded child maltreatment investigations and emergency medical care use based on hospital records.

**Results:**

Of the 4777 randomized families, 2327 were allocated to the intervention, and 2440 were allocated to services as usual. Among the children in the full study population, 2380 (49.8%) were female, 2397 (50.2%) were male, and 3359 (70.3%) were from racial/ethnic minority groups; of the 531 children included in the impact evaluation follow-up, 284 (53.5%) were female, 247 (46.5%) were male, and 390 (73.4%) were from racial/ethnic minority groups. Negative binomial models indicated that families assigned to FC had 39% fewer CPS investigations for suspected child maltreatment through 5 years of age (95% CI, −0.80 to 0.06; 90% CI, −0.73 to −0.01; control = 44 total investigations per 100 children and intervention = 27 total investigations per 100 children); intervention effects did not differ across subgroups. Families assigned to FC also had 33% less total child emergency medical care use (95% CI, −0.59 to −0.14; 90% CI, −0.55 to −0.18; control = 338 visits and overnight hospital stays per 100 children and intervention = 227 visits and overnight hospital stays per 100 children). Positive effects held across birth risk, child health insurance, child sex, single-parent status, and racial/ethnic groups. Effects were larger for nonminority families compared with minority families.

**Conclusions and Relevance:**

The findings of this randomized clinical trial suggest that, when implemented with high quality and broad reach, a brief postpartum nurse home visiting program can reduce population rates of child maltreatment and emergency medical care use in early childhood.

**Trial Registration:**

ClinicalTrials.gov Identifier: NCT01406184

## Key Points

**Question:**

What is the association of the Family Connects (FC) program, a universal newborn nurse home visiting program, with child maltreatment investigations and child emergency medical care use between birth and 5 years of age?

**Findings:**

In this randomized clinical trial, analyses of administrative records indicated that families assigned to FC had 39% fewer Child Protective Services investigations for suspected child abuse and neglect. Families assigned to FC also had a 33% decrease in total child emergency medical care use.

**Meaning:**

These findings indicate that, when implemented with high quality and broad reach, the FC program can have positive long-term benefits for population rates of child well-being.

## Teaser

This randomized clinical trial examines the effect of randomization to the Family Connects program on child maltreatment investigations and emergency medical care through 5 years of age.

## Introduction

Efforts to promote population health in early childhood remain a significant public health challenge in the United States. In 2019, almost 3.5 million children were subject to Child Protective Services (CPS) investigations for suspected maltreatment,^1^ and previous research suggests that children account for more than 28 million emergency department (ED) encounters annually.^2^ The risk is greatest for children from birth to 3 years, minority families, and families with low income. The Maternal, Infant, and Early Childhood Home Visiting (MIECHV) Program allocates $400 million annually to support the implementation of evidence-based home visiting models, providing more than 1 million home visits for children and families living in high-need communities. Although funding supports the dissemination of programs with demonstrated positive effects through randomized clinical trials (RCTs),^3^ the results do not always replicate when programs are scaled to serve larger and more diverse populations,^4,5^ highlighting the need for a framework for scaling and population impact.^6^

Family Connects (FC) is a MIECHV-approved,^7^ postpartum nurse home visiting program designed to reduce child maltreatment rates and improve health outcomes at a population level. Family Connects is a systems approach to supporting families, combining top-down engagement and alignment of community resources with bottom-up identification of family-specific needs through short-term nurse home visits for every birth in a community.^8^ Because home visits are offered to all families, no subsequent scaling is required, and no stigma is attached to participation, maximizing community acceptance at modest cost ($500-$700 per birth^9^).

Impact evaluation findings previously reported from the present RCT indicate that randomization to FC is associated with a variety of positive child and family outcomes, including increased connections to community resources, higher-quality home environments and parenting during infancy, reduced postpartum mental health symptoms, and a 37% reduction in child emergency medical care use through 24 months of age.^10,11^ Findings from a second, independent FC RCT identified multiple positive effects: increased community connections, reduced postpartum mental health symptoms, reduced emergency medical care use for infants with 1 or more medical risks at birth, and a 44% decrease in child maltreatment investigations through 24 months of age.^12^ Although these results indicate a consistent, positive pattern of benefits through the first 2 years of life, long-term child and family outcomes have yet to be investigated. This is an important lacuna, because the short-term benefits of many early childhood interventions are not sustained over time.^13^ In addition, rates of child maltreatment, the primary outcome for both FC RCTs, have not been examined in the present trial.

The primary goal of the present RCT is to examine the effect of FC on child maltreatment investigations and emergency medical care use through 5 years of age. A secondary goal is to examine whether program benefits for maltreatment and emergency medical care use differ across multiple child and family characteristics. It was hypothesized that (1) randomization to FC would be associated with lower rates of child maltreatment investigations and emergency medical care use and (2) moderation analyses would identify positive effects for all subgroups, but with larger effects for high-risk families that typically have worse outcomes but in FC received more intensive nurse interventions and connections to community services for long-term support.

## Methods

### Participants, Intervention Assignment, and Evaluation Design

Participants included families of all 4777 resident Durham County, North Carolina, births from 2 county hospitals between July 1, 2009, and December 31, 2010. As shown in Table 1, 2380 of all children born (49.8%) were female, 2397 (50.2%) were male, and 3359 (70.3%) were from racial/ethnic minority groups. All families randomly assigned to the FC program provided written informed consent for the intervention. A randomly selected subsample of 549 intervention and control group families (11.5% of the RCT population) participated in an independent outcome evaluation study, providing written informed consent for an in-home interview when the infant was 6 months of age and allowing study access to child hospital and maltreatment administrative records through 5 years of age. All study protocols were approved by the Duke Medicine institutional review board (trial protocol and statistical analysis plan in Supplement 1). This study followed the Consolidated Standards of Reporting Trials (CONSORT) reporting guidelines.

**Table 1.** Preintervention Sample Characteristics

Variable	RCT population and randomly selected and participating evaluation subsamples, No. (%)			RCT intervention and control populations, No. (%)		RCT intervention and control participating evaluation subsamples, No (%)	
	RCT population (n = 4777)	Randomly selected evaluation subsample (n = 664)	Participating evaluation subsample (n = 531)	RCT intervention population (n = 2327)	RCT control population (n = 2450)	Intervention evaluation subsample (n = 260)	Control evaluation subsample (n = 271)
Participation of selected	NA	NA	531 (80.0)	NA	NA	260 (81.3)	271 (78.8)
Child medical risk at birth							
Low birth weight	476 (10.0)	61 (9.1)	47 (8.9)	235 (10.1)	241 (9.8)	20 (7.8)	27 (10.0)
Gestation <37 wk	393 (8.2)	44 (6.7)	33 (6.3)	185 (7.9)	208 (8.5)	12 (4.7)	21 (7.8)
Any birth complications	354 (7.4)	38 (5.8)	32 (6.1)	175 (7.3)	179 (7.5)	10 (3.9)	22 (8.1)
Cesarean delivery	1460 (30.6)	210 (31.6)	169 (31.8)	718 (30.9)	742 (30.3)	84 (32.4)	85 (31.3)
Medicaid or no insurance	2904 (60.8)	419 (63.1)	348 (65.5)	1404 (60.4)	1499 (61.2)	165 (63.3)	183 (67.5)
Birthing parent age, mean (SD) y	28.5 (6.2)	28.5 (6.2)	28.3 (6.2)	28.5 (6.2)	28.5 (6.2)	28.2 (6.3)	28.4 (6.2)
Birthing parent race/ethnicity							
White, non-Hispanic	1418 (29.7)	194 (29.2)	141 (26.6)	686 (29.5)	732 (29.9)	74 (28.5)	67 (24.7)
Black	1754 (36.7)	252 (38.0)	209 (39.4)	833 (35.8)	921 (37.6)	95 (36.5)	114 (42.1)
Hispanic	1077 (22.6)	154 (23.2)	131 (24.7)	510 (21.9)	567 (23.1)	65 (25.0)	66 (24.4)
Other	528 (11.1)	64 (9.6)	50 (9.4)	298 (12.8)	230 (9.4)	26 (10.0)	24 (9.9)
Child female	2380 (49.8)	363 (54.7)	284 (53.5)	1137 (48.9)	1244 (50.8)	132 (50.8)	152 (56.1)

Abbreviations: NA, not applicable; RCT, randomized clinical trial.

**Figure 1.**
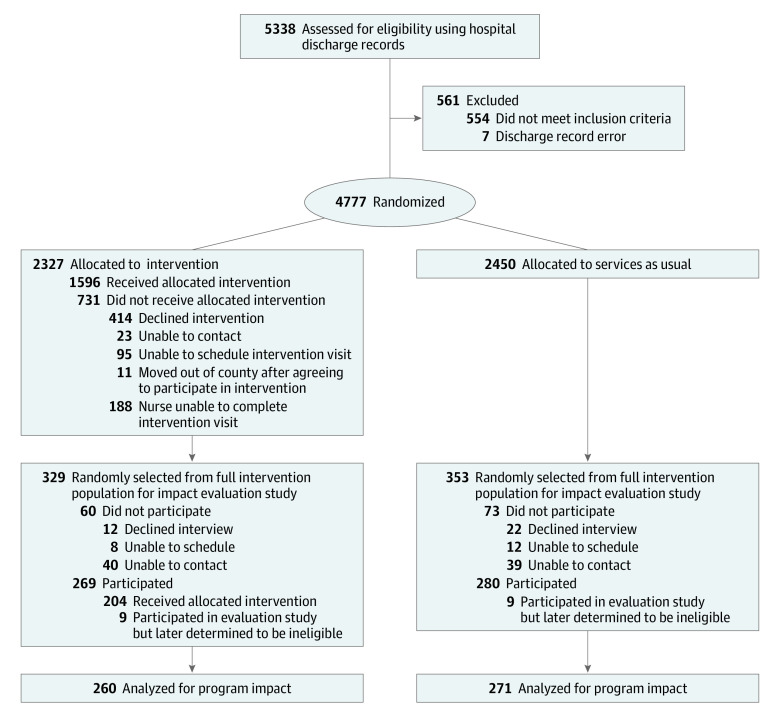
CONSORT 2010 Flow Diagram for Family Connects Randomized Clinical Trial Implementation and Evaluation

Discharge records for all county births at the 2 Durham County birthing hospitals were reviewed for possible inclusion. As shown in Figure 1, families of all 4777 resident births were randomly assigned a priori to intervention or treatment as usual based on infant birth date. Families of all 2327 even-date births were assigned to receive the FC program. Program staff attempted to engage and enroll all of these families in the intervention. Families of all 2450 odd-date births were assigned to receive treatment as usual. Control families were not offered the FC program but received all other services in the community, as usual. This approach allowed for the inclusion and evaluation of the implementation for all eligible families (not only families willing to participate in an RCT) with experimental rigor, without exception, and with ethical care for privacy.

Independent of the RCT implementation, investigators selected a random subsample of 549 families from the full population of 4777 families to participate in an impact evaluation study beginning when the infant was 6 months of age. Use of the random subsample allowed for an impact evaluation of a population-level intervention while maintaining feasible evaluation costs (eg, Moving to Opportunity for Fair Housing intervention^14^). Consistent with this evaluation strategy, a computer algorithm used electronic short-form birth records to randomly select 1 family per birth date for each day of the RCT implementation enrollment period (ie, from July 1, 2009, to December 31, 2010). Families that declined research participation were replaced with a randomly selected family with the same infant birth date, matched on birthing parent’s race/ethnicity to minimize possible sampling bias. Statistical power was estimated following the method of Cohen^15^ using 2-tailed tests with 0.80 power and a 0.05 significance level. Using techniques described by Stroup^16^ for power analyses with Poisson distributions, the study was sufficiently powered to detect a 27% decrease in the primary outcome.

Interviewers attempted to contact all families selected for impact evaluation follow-up, inviting them to participate in a research study examining associations between family use of community resources and child and family well-being. Families were asked to participate in an in-home interview when the infant was 6 months of age and to consent to administrative record review through 5 years of age. Families were blinded to the study goal of evaluating the FC program, and interviewers were blinded to FC program participation status. In total, 682 families were randomly selected, and 549 agreeed to participate (80.5%; 269 from the intervention group and 280 from the control group). After all families were enrolled and interviewed, a post hoc reconciliation of birth rosters from hospital discharge and public birth records identified 18 participating families (9 from the intervention group and 9 from the control group) lacking an accurate discharge record (13 without a discharge record, 3 with an incorrect birth date, and 2 with incorrect addresses affecting residency determination). These families were removed from the analyses (the final number familes was 531).

Study participation rates did not differ between intervention-assigned families (260 of 320 [81.3%]) and control-assigned families (271 of 344 [78.8%]). Intervention participation rates for the 260 FC program–assigned families were greater than those for the full population of intervention families (net complete, 204 of 260 [78.5%] vs 1596 of 2327 [68.8%]; *P* = .02). Among families participating in the FC program, dosage did not differ between the full population of intervention families (mean [SD] number of sessions, 1.52 [0.69]) and families participating in the impact evaluation study (mean [SD] number of sessions, 1.59 [0.71]) (*P* = .18). One participating child in the control group died at 12 months of age from a genetic condition. This child was retained in the analyses owing to the availability of partial outcome data; the inclusion or exclusion of this child does not meaningfully alter the results.

The baseline characteristics of the consenting families were collected from hospital discharge records and the in-home interview when the infant was 6 months of age. Table 1 provides these characteristics for the full birth population, the randomly selected group for evaluation, the group that consented for participation in the evaluation, the populations assigned to the intervention and control groups, and the participating intervention and control groups. These variables were used as covariates in analyses. Measures of the intervention impact were taken from administrative records.

### Intervention

The FC program adheres to a manualized protocol^8^ consisting of the following 3 core components: (1) the direct intervention consists of engagement with families shortly after birth, (2) assessment and intervention with families through 1 to 3 home visits with a registered public health nurse, and (3) a final telephone contact 4 weeks after the home visit(s). During in-home visits, nurses provide brief educational interventions for all families (eg, feeding practices) and, using a high-inference approach combining parent self-report, direct observation, and nurse clinical judgment, systematically assess family need across 12 empirically derived factors associated with child health and well-being (health care: maternal health, infant health, and health care plans; parenting and childcare: childcare plans, parent-child relationship, and management of infant crying; family material resources and safety: material supports, family and community safety, and birthing parent history of parenting difficulties; and parent well-being: mental health, substance abuse, and social-emotional support).

Nurses address family needs based on their ratings of each factor on a 4-point scale: 1 indicates low risk, and there is no subsequent intervention; 2 indicates moderate risk, with short-term, nurse-delivered education; 3 indicates significant risk, with collaborative connections to community services and resources tailored to address particular needs (eg, postpartum depression treatment, food assistance, and long-term home visiting program); and 4 indicates imminent risk, with emergency intervention (<1% of cases). With family consent, summary reports are sent to parent and infant health care professionals to support medical home connections. The final telephone contact 4 weeks after case closure ascertains family-consumer satisfaction and confirms outcomes for community referrals.

The second core component is a comprehensive community alignment process that includes engagement of key community services and stakeholders, creation of a comprehensive electronic directory (“agency finder”) of community resources (allowing home visiting nurses to match services to family need), and a community-program “feedback loop” with bidirectional communication supporting alignment of community services. The third component is a comprehensive electronic database that houses the electronic agency finder and serves as the clinical record of the intervention.

As reported by Dodge et al,^10^ 80.1% of all eligible families (1863 of 2327) scheduled an FC home visit; 85.7% of these families (1596 of 1863) successfully completed the program (net completion rate, 68.6%). Nurse adherence to the home visit protocol was 83.6% (5267 of 6304 program elements checked); interrater agreement across all 12 risk factors was substantial (κ = 0.69).^15^ Of 1596 families, 50 (3.1%) stopped assessment because of family choice. A total of 1453 of 1546 families (94.0%) had 1 or more nurse-identified risks targeted for intervention, 681 of 1546 families (44.0%) had 1 or more risks best addressed by community connections, and 579 of 730 referred families (79.3%) reported at least 1 successful community connection during the follow-up telephone contact 4 weeks later.

### Covariates

Hospital administrative records were coded for the presence of child medical risks at birth (any of: birth weight, <2500 g; gestational age, <37 weeks; and any *International Classification of Diseases, Ninth Revision* codes indicating birth complications or trauma^17^) and child sex (0 = boy and 1 = girl). Single-parent household status (0 = no and 1 = yes), child Medicaid or no health insurance status (0 = no and 1 = yes), and self-identified race/ethnicity (0 = nonminority and 1 = minority) were coded from maternal report.

### Outcome Measures

As in the pretrial registry, the primary study outcome was CPS reports for suspected maltreatment. State administrative records were examined through 5 years of age for the 531 evaluation study families. Records were coded for the total number of investigations for suspected maltreatment per child*.* Records were also coded for the total number of substantiated per-child investigations if evidence of maltreatment was confirmed; this outcome was not tested because only 10 of 531 families (1.9%) had 1 or more substantiations through 5 years of age. The low base rate is attributed to reforms in state child welfare laws that prioritized engaging investigated families in clinical services rather than substantiating maltreatment.^18^

Hospital administrative billing records were examined through 5 years of age and coded for the total number of per-child ED visits and overnight stays in hospital. The 2 scores were summed to measure total per-child emergency medical care use.

### Missing Data

Four of the 531 evaluation study participants (0.8%) had at least 1 missing value, representing 0.2% of all data points (12 of 6903). Following guidance by Schafer and Graham,^19^ single imputation was used to account for missing values from other family and demographic characteristics prior to estimating intervention effects.

### Statistical Analysis

Statistical analysis was conducted from November 6, 2020, to April 25, 2021. Analysis was conducted on an intent-to-treat basis. We used SAS, version 9.4 (SAS Institute Inc) to estimate the effect of randomizaton to the FC program (or not) on CPS child maltreatment investigations and child emergency medical care use, regardless of intervention participation or adherence. Negative binomial regression models were used because the maltreatment and emergency medical care outcomes were count variables with skewed distributions.^20^ First, main effect models were estimated with infant birth risk, child health insurance status, birthing parent race/ethnicity, single-parent status, and child sex as covariates. Next, moderation analyses were estimated to examine whether intervention effects differed based on child and family characteristics. Moderators were examined individually with all covariates included in the model; a Holm-Bonferonni sequential correction was applied to account for increased type I error risk resulting from 20 individual moderation tests.^21^ Post hoc tests of significant interactions remaining after correction were conducted following the method of Aiken and West.^22^ Results are reported with 2-tailed *P* values and 95% and 90% CIs, with *P* < .10; a percentage decrease is reported as ([count per 100 control children − count per 100 intervention children]/count per control children) ×100.

## Results

Among the children in the full study population, 2380 (49.8%) were female, 2397 (50.2%) were male, and 3359 (70.3%) were from racial/ethnic minority groups; of the 531 children included in the impact evaluation follow-up, 284 (53.5%) were female, 247 (46.5%) were male, and 390 (73.4%) were from racial/ethnic minority groups (Table 1). A total of 46 of 260 children (17.7%) in the intervention group and 59 of 271 children (21.8%) in the control group were the subject of 1 or more CPS investigations; 151 of 260 children (58.1%) in the intervention group and 193 of 271 children (71.2%) in the control group experienced 1 or more emergency medical care encounters. Descriptive statistics for child maltreatment investigations and emergency medical care, by intervention group and subgroup, are presented in Table 2.

**Table 2.** Descriptive Statistics for Child Maltreatment Investigations and Emergency Medical Care Use Through 5 Years of Age by Treatment Group and Subgroup

Group	Child maltreatment investigations		Child emergency medical care use	
	Total No. of investigations/100 children	Mean (SD)	Total No. of visits and overnight hospital stays/100 children	Mean (SD)
Overall				
All families–I (n = 260)	27	0.27 (0.77)	227	2.27 (3.34)
All families–C (n = 271)	44	0.44 (1.05)	338	3.38 (5.49)
Child medical risk at birth				
No medical risk–I (n = 229)	29	0.29 (0.81)	228	2.28 (3.35)
No medical risk–C (n = 232)	39	0.39 (0.95)	275	2.75 (4.01)
Medical risk–I (n = 31)	10	0.10 (0.40)	223	2.23 (3.33)
Medical risk–C (n = 39)	74	0.74 (1.52)	713	7.13 (9.98)
Child insurance				
Private insurance–I (n = 96)	5	0.05 (0.27)	73	0.73 (1.70)
Private insurance–C (n = 94)	1	0.01 (0.10)	148	1.48 (4.01)
Medicaid or no insurance–I (n = 164)	40	0.40 (0.93)	318	3.18 (3.72)
Medicaid or no insurance–C (n = 177)	67	0.67 (1.24)	440	4.40 (5.90)
Birthing parent race/ethnicity				
Nonminority–I (n = 75)	11	0.11 (0.39)	96	0.96 (1.98)
Nonminority–C (n = 69)	10	0.10 (0.60)	217	2.17 (5.19)
Minority–I (n = 185)	34	0.34 (0.88)	281	2.81 (3.63)
Minority–C (n = 202)	56	0.56 (1.15)	380	3.80 (5.54)
Single-parent status				
Partnered–I (n = 165)	13	0.13 (0.70)	147	1.47 (2.60)
Partnered–C (n = 156)	20	0.20 (0.71)	218	2.18 (3.90)
Single parent–I (n = 95)	51	0.51 (0.84)	366	3.66 (3.99)
Single parent–C (n = 115)	77	0.77 (1.32)	502	5.02 (6.78)
Child sex				
Girl–I (n = 129)	34	0.34 (0.98)	178	1.78 (2.66)
Girl–C (n = 151)	44	0.44 (1.03)	275	2.75 (3.93)
Boy–I (n = 131)	20	0.20 (0.49)	276	2.76 (3.85)
Boy–C (n = 120)	45	0.45 (1.08)	418	4.18 (6.92)

Abbreviations: C, control group; I, intervention group (assigned to Family Connects program).

### Intervention Effect on Child Maltreatment Investigations

Results from negative binomial models indicated that random assignment to FC was associated with a 39% decrease in mean total per-child CPS investigations for suspected maltreatment (95% CI, −0.80 to 0.06; 90% CI, −0.73 to −0.01; control = 44 total investigations per 100 children, intervention = 27 total investigations per 100 children) (Table 3). Results from moderation models indicated that intervention effects on total maltreatment investigations did not differ across subgroups.

**Table 3.** Main Effect Analyses Examining Family Connects Effect on Total Child Maltreatment Investigations and Emergency Medical Care Use Through 5 Years of Age (N = 531)

Variable	Maltreatment investigations from birth to 5 y			Emergency medical care use from birth to 5 y		
	90% CI	95% CI	*P* value	90% CI	95% CI	*P* value
Child medical risk at birth^a^	−0.43 to 0.59	−0.53 to 0.69	.80	0.40 to 0.93	0.35 to 0.98	<.001
Child Medicaid or no health insurance^b^	1.63 to 3.19	1.48 to 3.34	<.001	0.85 to 1.37	0.80 to 1.42	<.001
Mother minority status^c^	−0.54 to 0.71	−0.66 to 0.83	.82	−0.33 to 0.22	−0.38 to 0.27	.75
Mother single parent status^d^	0.44 to 1.18	0.37 to 1.25	<.001	0.29 to 0.70	0.25 to 0.74	<.001
Child sex^e^	−0.13 to 0.58	−0.20 to 0.65	.30	−0.54 to −0.17	−0.58 to −0.13	.002
Treatment^f^	−0.73 to −0.01	−0.80 to 0.06	.09	−0.55 to −0.18	−0.59 to −0.14	.001

^a^ Child medical risk at birth: 0 = no and 1 = yes.

^b^ Child Medicaid or no health insurance: 0 = no and 1 = yes*.*

^c^ Mother minority status: 0 = nonminority and 1 = minority.

^d^ Mother single parent status: 0 = no and 1 = yes.

^e^ Child sex: 0 = boy and 1 = girl.

^f^ Treatment: 0 = control group and 1 = intervention group (assigned to Family Connects program).

### Intervention Effect on Emergency Medical Care

**Figure 2.**
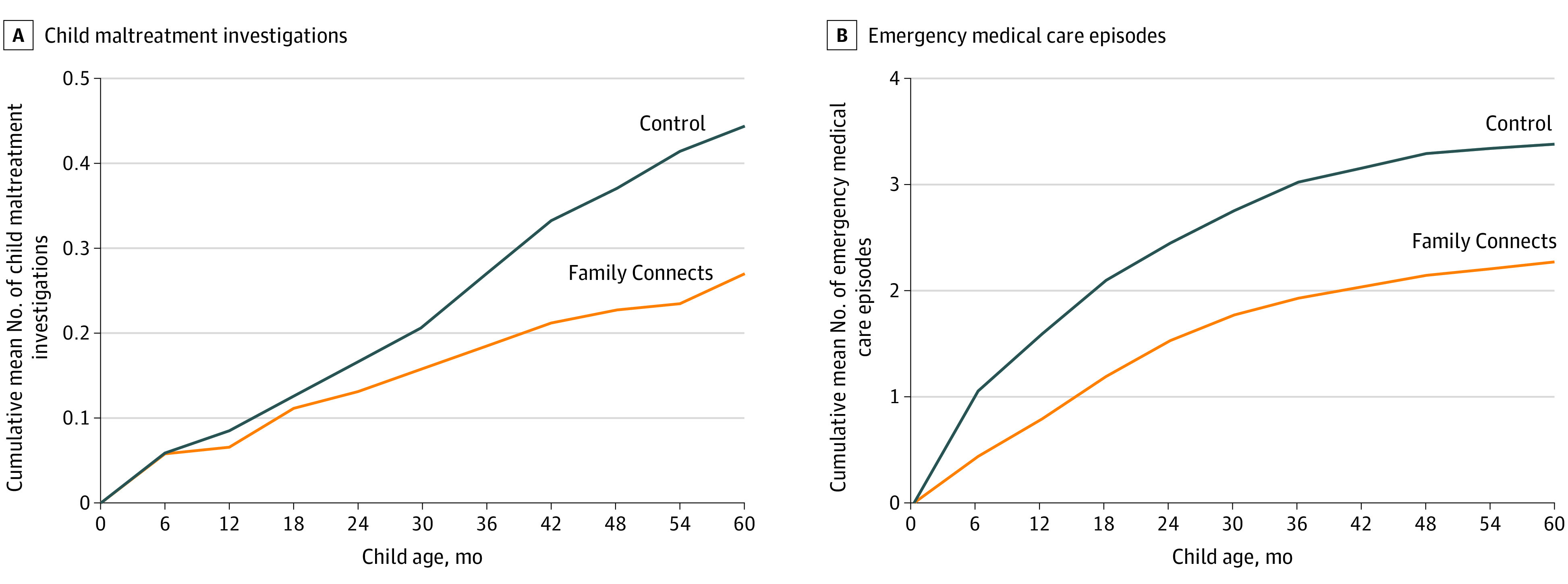
Cumulative Mean Number of Child Protective Services Investigations and Emergency Medical Care Episodes From Birth to 60 Months of Age, by Intervention Group (N = 531)

The results from negative binomial models indicated that randomization to the FC program was associated with a 33% decrease in the mean total per-child emergency medical care use (95% CI, −0.59 to −0.14; 90% CI, −0.55 to −0.18; control = 338 visits and overnight hospital stays per 100 children, intervention = 227 visits and overnight hospital stays per 100 children) (Figure 2).

The results from moderation models indicated that intervention effects differed based on racial/ethnic majority or minority status of parent. Post hoc analyses revealed a positive effect of intervention assignment for both nonminority and minority families but a larger decrease for nonminority families. Among nonminority families, randomization to the FC program was associated with a 1.51-unit mean decrease in the mean total use of emergency medical care (95% CI, −1.68 to −0.32; 90% CI, −1.57 to −0.43; control = 217 visits and overnight hospital stays per 100 children, intervention = 96 visits and overnight hospital stays per 100 children) compared with control group nonminority families. Randomization to the FC program was also associated with a 0.99-unit decrease in the mean total use of emergency medical care for minority families (95% CI, −0.43 to 0.03; 90% CI, −0.39 to −0.01; control = 380 visits and overnight hospital stays per 100 children, intervention = 281 visits and overnight hospital stays per 100 children).

Examining each component of total emergency medical care individually, we found that randomization to the FC program was associated with a 17% decrease in the mean total number of ED visits (−0.38 to 0.03; 90% CI, −0.35 to −0.002; control = 243 visits per 100 children, intervention = 202 visits per 100 children) and a 73% decrease in the mean total number of hospital overnight stays (95% CI, −1.89 to −0.14; 90% CI, −1.75 to −0.28; control = 95 overnight stays per 100 children, intervention = 25 overnight stays per 100 children). No significant interaction effects were observed for either construct.

## Discussion

Study results demonstrate that random assignment to receive short-term, universal postpartum home visits from a nurse is associated with reduced child maltreatment rates and emergency medical care use through 5 years of age. Random assignment to the FC program was associated with a 39% decrease in the mean number of CPS investigations for suspected maltreatment and a 33% decrease in the mean rate of emergency medical care use. To our knowledge, these findings are the first reported on the 5-year effect of early home visiting on a community-wide population.

The effect of randomization to the FC program held across every subgroup tested, including families with high and low child medical risk at birth, Medicaid or no health insurance and private insurance, single-parent and 2-parent families, racial/ethnic nonminority and minority status, and child sex. These findings of reduced rates of maltreatment investigations and emergency medical care use support the value of offering the FC program universally. Unlike some programs that target specific demographic subgroups, the FC program features community-wide implementation; these findings support that policy decision. The effect on child maltreatment investigations was observed at *P* < .10, rather than the typical *P* < .05, likely owing to lower base rates than anticipated before the trial. Nevertheless, the observed 39% decrease in the mean number of CPS investigations for suspected maltreatment is practically important for efforts to promote community-wide maltreatment prevention. In addition, the magnitude of the effect on emergency medical care was robust for both racial/ethnic minority and nonminority families, but larger for nonminority families compared with minority families. It is unclear why this racial/ethnic difference emerged; future studies should attempt understand both the reasons for the difference and whether similar patterns occur across diverse minority populations.

### Limitations

This study has some limitations. This evaluation was conducted in only 1 medium-sized urban community with high socioeconomic diversity; findings may or may not be generalizable to communities that differ markedly in size, community resources, sociodemographic characterisitcs, or base rates of child maltreatment and emergency medical care use. We suggest similar investigations of the effect in other communities.

Findings from a quasi-experimental field study in 4 low-resource, rural communities found that the FC program was associated with multiple short-term positive benefits for families during infancy, including reductions in emergency medical care use^23^; however, the effects on child maltreatment are yet to be examined. Like the present study, the FC program was implemented with high quality in these rural communities. It is not clear whether the positive effect would hold in contexts of lower-quality implementation. In addition, although the findings suggest possible positive returns for communities through avoidance of costly outcomes, a comprehensive study of the benefits and costs of the FC program is needed.

Another limitation applies only to the outcomes studied here (child maltreatment and emergency medical care use); it is not known whether the 5-year effects of the FC program hold for the other outcomes found at 6 months of age, including community connections, mental health, and parenting. Although data on emergency medical care use were collected from both county hospitals, some families may have sought care elsewhere. The choice of care should not differ based on intervention assignment; the FC program neither endorses nor directs families toward specific medical organizations. Finally, the program completion rate among eligible families participating in the evaluation study was greater than that among eligible families in the full population (78% vs 69%). The present results may represent a high-end estimate of full-population effects.

## Conclusions

This study found that, when implemented with high quality and broad reach, the FC program is associated with reduced rates of child maltreatment investigations and emergency medical care use through 5 years of age. These findings argue that such a public health prevention approach has benefits that extend throughout early childhood, making such programs a worthy community investment.

## Notes

Supplement 1. Trial Protocol and Statistical Analysis Plan

Supplement 2. Data Sharing Statement
