# Supplementary material for: Effect of a Universal Postpartum Nurse Home Visiting Program on Child Maltreatment and Emergency Medical Care at 5 Years of Age: A Randomized Clinical Trial
Source: JAMA Netw Open. 2021 Jul 7;4(7):e2116024. doi: 10.1001/jamanetworkopen.2021.16024 (PMC8264647; doi:10.1001/jamanetworkopen.2021.16024)
Supplement: Supplement 1. — Trial Protocol and Statistical Analysis Plan [file jamanetwopen-e2116024-s001.pdf]

eIRB DukeMedicine Institutional Review Board for Clinical Investigations

Date: Tuesday, August 27, 2019 1:07:22 PM
View: 01. Study Title and Research Personnel

Print Close

01. Study Title and Research Personnel

User Guide

* **Short Title:**
Durham Connects Evaluation

* **Full Title:**
Durham Connects: Evaluation of a Universal Nurse Home Visiting Program

* **Study Organization:**
Social Science Research Institute

CRU (Clinical Research Unit) or Oversight Organization Selection:

* Is Duke University Hospital, Durham Regional Hospital, Duke Raleigh Hospital, any Duke Clinic or any other Duke Medicine entity a site where interventions or interactions with research subjects will occur as part of this study?
Yes **No** (selected)

* Will a Duke faculty or staff member be involved with interventions, observations, surveys or interactions with Duke patients?
Yes **No** (selected)

* Does this study involve the use of biological specimens from Duke patients?
Yes **No** (selected)

* Does this study involve access to confidential, private information from Duke patients?
Yes (selected) No

* Does this study require CRU oversight for any other reason not listed above?
Yes **No** (selected)

Note: Only people with current Human Subjects Protection (HSP) certification appear on the Select Person lists. If you cannot find the person, go to the FAQ section of the eIRB Home page and click the link *I'm trying to add someone to my study, but I can't find their name.*

* **Principal Investigator:**
Kenneth Dodge

Primary Study Coordinator (CRC):

Primary Regulatory Coordinator:
William Goodman

Co-Principal Investigators:

First Name	Last Name	Department
There are no items to display		

Other Key Personnel:

	Name of Individual	Role on study	Edit Rights	Receive Email	CITI Expiration
View	Yu Bai	Statistician	no	no	12/2/2018
View	Matthew Edwards	Computer Programmer	no	no	3/28/2021
View	William Goodman	Investigator	yes	yes	5/8/2020
View	Robert Murphy	Investigator	yes	no	2/22/2020
View	Philip Nousak	Data Manager	no	no	12/14/2019
View	Karen O'Donnell	Investigator	yes	no	1/14/2021

View: 02. Study Personnel Outside Duke

02. Study Personnel Outside Duke

User Guide

For all Key Personnel who are not Duke employees, complete and attach an Outside Duke Key Personnel Form.

Document Name	Date Created	Last Modified	Revision
Human Subjects Training D Daro	11/23/2009 12:12 PM	11/23/2009 12:12 PM	0.01
Human Subjects Training L Huang	11/23/2009 12:12 PM	11/23/2009 12:12 PM	0.01
Outside Personnel Form	11/23/2009 12:12 PM	11/23/2009 12:12 PM	0.01
Univ of Chicago IRB Approval	11/24/2009 10:31 AM	11/24/2009 10:31 AM	0.01
Univ of Chicago IRB Protocol	11/24/2009 10:31 AM	11/24/2009 10:31 AM	0.01

View: 03. Protocol Application Type

03. Protocol Application Type

User Guide

* **Select the type of protocol you are creating:**

- ○ **Regular Study Application**
  Most common. The IRB will determine if the study is eligible for expedited review or requires full board review upon submission.
- ◉ **Application for Exemption from IRB Review**
  Includes Exempt (45 CFR 46.101 (b)), Not Human Subject Research (45 CFR 46.102 (f)), & Not Research (45 CFR 46.102 (d)).
- ○ **External IRB Application**
  Includes phase II, phase III, phase IV protocols that are industry sponsored multi-site studies, and includes selected DCRU phase I protocols.
- ○ **Trainee Research While Away from Duke**
  Research conducted by medical students overseen by the Office of Curriculum & other student/trainee research away from Duke.
- ○ **Emergency Use of a Test Article**
  Emergency use of an investigational drug or biologic, emergency use of an unapproved device.

View: 03.1 Exempt Project Summary

03.1 Exempt Application

User Guide

Project Summary:

1. Provide a description of the research that includes the background, objectives and conduct of

the study (procedures).
2. Describe the subject population (include number of subjects, age range, and recruitment methods) or the materials (records or specimens and their source) to be used in this study.
3. Specify the risks and benefits.
4. Describe the data collection and confidentiality.
5. Describe the consent process. Append a consent form, if appropriate.

Attach Project Summary:

Name	Date Modified
Emails with John Falletta	5/21/2009 11:22 AM
IRB Data Center Memos CO ME.pdf	11/2/2009 2:58 PM
IRB Investigator Memo - CC.pdf	11/2/2009 2:58 PM
IRB Investigator Memo - KA.pdf	11/2/2009 2:58 PM
IRB Investigator Memo - KD.pdf	11/2/2009 2:58 PM
IRB Investigator Memo KOD.txt	11/2/2009 2:58 PM
IRB InvestigatorMemo RM.txt	11/2/2009 2:59 PM
Durham Connects Evaluation - Research Summary (Revised 20150413).doc	4/13/2015 9:40 PM

Is this activity "research involving human subjects" as defined in 45 CFR part 46.102?

Yes **No** (selected)

If Yes, select any approprate categories and explain in the text box below:

Criteria

There are no items to display

Explanation:

View: 18. End of Application Form

End of Application Form

You have reached the end of the New Protocol Application form. Upon clicking the "Finish" button below, this application **will not** automatically be submitted for review. It will instead appear under the "Presubmission" tab on your workspace, allowing further edits to be made to the application later if it is not yet ready for submission.

If this application is complete and ready to be submitted for review, you must click the "Submit Study" activity button, located in the left column of this application's workspace, to begin the Duke HRPP review process.

Statistical Analysis Plan (SAP)

DURHAM CONNECTS RCT EVALUATION

Principal Investigator	Kenneth A. Dodge, PhD<br>William McDougall Professor of Public Policy<br>Sanford School of Public Policy<br>Professor of Psychology and Neuroscience<br>Duke University<br>Durham, NC
Protocol identification number	Pro00020974 (Durham Connects Evaluation; Approved 12/17/2009)
ClinicalTrials.gov identifier	NCT01406184
Authors	Yu Bai, PhD<br>Statistician III<br>Center for Child and Family Policy<br>Duke University<br>Durham, NC<br><br>W. Benjamin Goodman, PhD<br>Research Scientist<br>Center for Child and Family Policy<br>Duke University<br>Durham, NC
Version	1.1

SIGNATURE PAGE

Kenneth A. Dodge 01-06-2021

Principal Investigator

Kenneth A. Dodge Date

Abbreviations

CPS Child Protective Services

RCT Randomized Controlled Trial

Table of Contents

ABBREVIATIONS **2**
1. INTRODUCTION **5**
2. STUDY DESIGN **5**
2.1 SAMPLE SIZE CALCULATION 6
3. AIMS AND OBJECTIVES **7**
4. OUTCOMES **8**
4.1 PRIMARY OUTCOME 8
4.2 SECONDARY OUTCOMES 8
5. POPULATIONS AND SUBGROUPS TO BE ANALYZED **9**
5.1 POPULATIONS 9
5.2 SUBGROUPS 10
6. ANALYSES **10**
6.1 PRIMARY OUTCOME 11
6.2 SECONDARY OUTCOMES 11
7. MISSING DATA **11**

1. Introduction

The aim of the current study is to test random assignment to the Durham Connects (now called Family Connects) community-wide newborn nurse home visiting program on: (1) child maltreatment assessments and substantiations, (2) mother and infant health and health care utilization, (3) parenting and parent-child relationship quality, and (4) family connections to community resources.

This statistical analysis plan (SAP) will give more detailed descriptions of the outcomes in the study and the corresponding analyses.

2. Study Design

Study subjects include all resident Durham County births from July 1, 2009 – December 31, 2010 at two county birthing hospitals (Duke University Hospital and Durham Regional Hospital).

The study is a two-armed, parallel-design RCT. Families of infants born during the RCT enrollment period were randomized to be eligible to receive the Durham Connects home visiting intervention or to the control group. Families were randomized *a priori* to one of the two intervention groups based on infant birth date: 1) *even birth date* families were assigned to receive Durham Connects; program staff attempted to engage all of these families and schedule a home visit; 2) *odd birth date* families were not offered Durham Connects but received other community services as usual and served as the control group. Although differing from traditional randomization procedures in clinical trials, whereby individuals are randomized after providing informed consent, the *a priori* randomization procedure utilized in the current trial was necessary to examine program implementation and impact within the full community population (not only those families willing to participate in a randomized trial). This approach allowed for inclusion of all eligible families (i.e., families living in Durham County giving birth at Duke University Hospital) with experimental rigor, and without exception, but with ethical care for privacy.

To examine impact of assignment to intervention, a separate research evaluation design was implemented independently by a team unaffiliated with the intervention, beginning at age 6 months. A random, representative subsample of families was selected by computer algorithm using electronic short-form birth records to participate in the impact evaluation study. Research assistants blind to experimental condition tried to locate all randomly selected families and to solicit consent to participate in a research study of infant development. A total of 549 families, representing one birth for each day of the RCT implementation enrollment period, were consented and enrolled in the study. The study design is presented in Figure 1 below.

Figure 1.

CONSORT 2010 Flow Diagram for Family Connects RCT Implementation and Evaluation

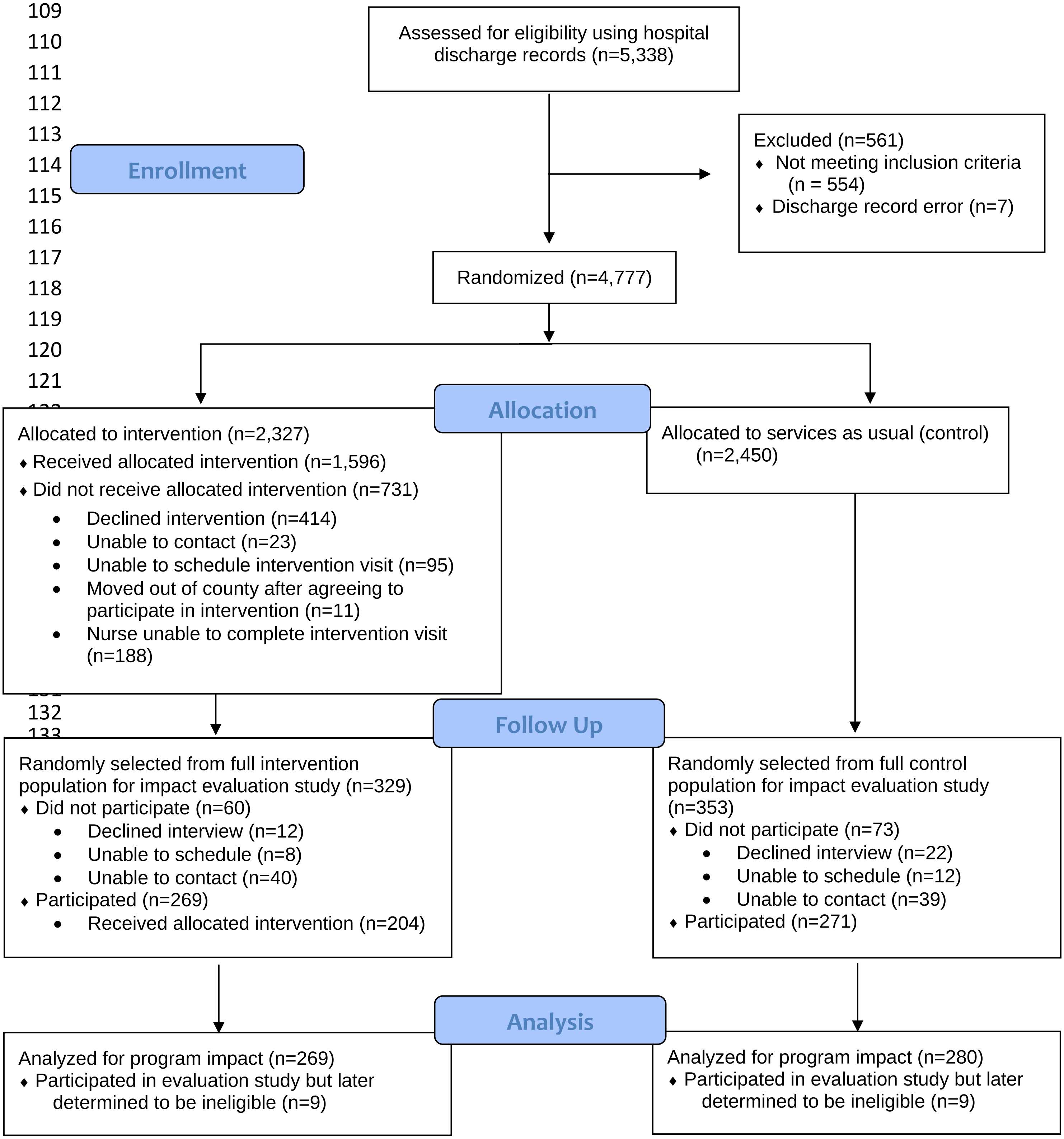

2.1 Sample Size Calculation

We conducted a series of analyses to estimate the statistical power required to detect significant differences between program and control groups for our key outcomes. Following Cohen (1988), all analyses estimated at least .80 power and a significance level of .05. Using *Gpower* software (Faul, Erdfelder, Lang, & Buchner, 2007) and conservative estimates of program effects, we found the study to be adequately powered to test our principal hypotheses. For example, in the domain of maltreatment, we assessed power for mothers' self-reported neglectful behavior (PC-CTS). To estimate power, we drew on pilot data collected by the Durham Family Initiative in low-income/high DSS neighborhoods in Durham (*N* = 191). As described earlier, the self-reported rate of neglectful behavior among mothers of children under 12 months of age was 25.7%. For our Durham community sample, we expect the rate of neglectful behavior to be considerably lower –15.4%. We further expect that the Durham Connects program will reduce this rate by half – to 7.7% (ES = .15, small to medium effect). With a sample of 500, we will have .91 power to detect an effect of reducing maltreatment from .16 to .08. In the area of child health, we estimated power to affect immunization rates in Durham. According to the Durham County Health Department, the percent of children under age 2 in Durham who are up-to-date on immunizations is 47%. We expect that Durham Connects will increase this rate by 25% to 59% (ES = .17, small to medium effect). With a sample of 500 available for pediatric chart review, we will have .96 power to detect an effect of this magnitude. Last, we will test the extent to which program effects on maltreatment and child outcomes are mediated by (a) improved family service receipt, (b) connection to medical home, and (c) enhancements in maternal self-efficacy. According to Fritz and MacKinnon's criteria, at .80 power, in order to detect a small-to-medium-sized effect (parameter value = .26) of the program on a hypothesized mediator (e.g., maternal self-efficacy), and a small-to-medium-sized effect of the mediator on the outcome (e.g., maltreatment, child health) adjusted for program effects, a total sample size of 161 would be required. Thus, the proposed sample of 500 will provide ample power to detect small-to-medium-sized mediated effects.

3. Aims and Objectives

To examine whether random assignment to receive a brief, postpartum nurse home visiting program predicts (1) reductions in child maltreatment assessments and substantiations, (2) increases in mother and infant health and decreases in mother and infant emergency medical care utilization, (3) higher quality parenting behaviors and parent-child relationship quality, and (4) increased family connections to community resources.

4. Outcomes

This section will present the outcomes investigated to answer the study aims and objectives. The analyses are described in Section 6.

4.1 Primary Outcome

1. **Child maltreatment assessments and substantiations**. This outcome will be measured using CPS administrative records from the North Carolina Division of Social Services. Records will be collected beginning at birth and continue through child age 12 years.

4.2 Secondary Outcomes

1. **Child emergency room (ER) presentation rates**. This outcome will be measured using Duke University Health System administrative records of all outpatient emergency room presentations for the study child. Records will be collected beginning at birth and continue through child age 12 years.

2. **Child hospital overnight stays**. This outcome will be measured using Duke University Health System administrative records of all inpatient nights spent in hospital for the study child. Records will be collected beginning at birth and continue through child age 12 years.

3. **Mother emergency room (ER) presentation rates**. This outcome will be measured using Duke University Health System administrative records of all outpatient emergency room presentations for the study mother. Records will be collected beginning at birth of the study child and continue through child age 12 years.

4. **Mother hospital overnight stays**. This outcome will be measured using Duke University Health System administrative records of all inpatient nights spent in hospital for the study mother. Records will be collected beginning at birth of the study child and continue through child age 12 years.

5. **Mother postnatal well-care compliance.** This outcome will be measured based on mother self-report of completing (or not) her 6-week postpartum health exam. This outcome will be measured during an in-home interview when the study child is approximately 6 months old.

6. **Child postnatal well-care compliance.** This outcome will be measured based on mother self-report of having taken (or not) her child for a pediatric well-child exam within the past month. This outcome will be measured during an in-home interview when the study child is approximately 6 months old.

7. **Mother mental health.** This outcome will be measured based on mother self-report on two brief questionnaires: the Edinburgh Postnatal Depression Scale (Cox, Holden, & Sagovsky, 1987; assessing postpartum depression) and the General Anxiety Disorder – 7 (Spitzer, Kroenke, Williams, & Lowe, 2006; assessing generalized anxiety). This outcome will be measured during an in-home interview when the study child is approximately 6 months old.

8. **Mother parenting behaviors.** This outcome will be measured based on mother self-report of positive and negative parenting behaviors toward the study child (Durham Family Initiative, 2008; Lounds, J.J., Borkowski, J.G. & Whitman, T.L., 2004; Straus, M. A., Hamby, S. L., Finkelhor, D., & Runyan, D., 1995). This outcome will be measured during an in-home interview when the study child is approximately 6 months old.

9. **Mother infant intentionality beliefs.** This outcome will be measured based on mother self-report regarding the extent to which infants can intentionally engage in negative behaviors (Feldman & Reznick, 1996). This outcome will be measured during an in-home interview when the study child is approximately 6 months old.

10. Father-child relationship quality. This outcome will be measured based on mother report of father involvement in caring for the study child (Center for Research on Child Wellbeing, 2008). This outcome will be measured during an in-home interview when the study child is approximately 6 months old.

11. **Family connections to community services and resources.** This outcome will be measured based on mother self-reported family use (or not) of various formal and informal services and resources in the Durham, NC community. This outcome will be measured during an in-home interview when the study child is approximately 6 months old.

12. **Out of home childcare utilization.** This outcome will be measured based on mother self-reported use (or not) of out-of-home childcare for the study child. This outcome will be measured during an in-home interview when the study child is approximately 6 months old.

5. Populations and Subgroups to be Analyzed

5.1 Populations

1. **Intent-to-treat (ITT).** All randomized study subjects that provided written consent to participate in the outcome evaluation interview at infant age 6 months. To avoid potential contamination of the study design, any family that had previous participated in Durham

Connects prior to the start of this RCT were removed from the sample. Additionally, to avoid violating assumptions of linear regression models, for participating mothers who gave birth to multiples (e.g., twins), one child was randomly chosen for inclusion in the study analyses.

5.2 Subgroups

Five sets of subgroup analyses will be conducted based on preliminary analyses that suggest baseline differences exist between the intervention and control groups based on presence or absence of multiple demographic or medical risk factors.

1. **Infant total medical risk at birth.** Subgroup analyses will examine differences in outcomes based on the total number of medical risks at birth, a 0-3 count variable summing the following: 1) born at less than 2500 grams; 2) less than 37 weeks gestational age, and 3) birth complications, not specified.

2. **Mother and infant health insurance.** Subgroup analyses will examine differences in outcomes based on whether the mother and infant have 1) Medicaid or no health insurance; or 2) private health insurance.

3. **Mother race and ethnicity.** Subgroup analyses will examine differences in outcomes based on mother race and ethnicity, coded as 1) minority; or 2) White.

4. **Mother single parent status.** Subgroup analyses will examine differences in outcomes based on mother single parent status, coded as 1) single parent; or 2) not a single parent.

5. **Infant/child gender.** Subgroup analyses will examine differences in outcomes based on child gender, coded as 1) male; 2) female.

6. Analyses

Descriptive statistic (mean, standard deviation, frequency, and percent) will be used to describe the birth risks, demographic and socioeconomic characteristics, and primary and secondary outcomes for the whole sample. Then, the birth risks and demographic and socioeconomic characteristics at baseline will be compared between children and their mothers in the treatment group and those in the control group by using chi-squared tests for categorical variables and using t-tests for continuous variables. If any imbalance at baseline exists between the two groups, a regression adjustment will be used to create adjusted means in outcomes for each group by controlling birth risks and demographic and socioeconomic characteristics. Stata 14.2 and SAS 9.4 will be used for all statistical analysis.

6.1 Primary Outcome

A negative binomial model will be applied when the outcomes are number of assessments and substantiations. The independent variable is treatment status (treatment=1). The covariates include birth risks, demographic, and socioeconomic characteristics. An adjusted mean of the outcomes in each group will be reported as well as a group difference and its confidence interval.

6.2 Secondary Outcomes

A linear model will be applied if the outcomes are continuous variables. A negative binomial model will be applied if the outcomes are count variables. A logistic model will be applied if the outcomes are dichotomous variables. The covariates include birth risks, demographic, and socioeconomic characteristics. An adjusted mean of the outcomes in each group will be reported as well as a group difference and its confidence interval.

7. Missing Data

Missing data issue is commonly seen in social science research. Our missing data could come from item nonresponse and missingness in administrative data and survey. Single and multiple imputation techniques can reduce non-response bias, improve efficiency, and increase statistics power in parameter estimates, as compared to listwise deletion. Consistent with guidelines established by Schafer and Graham (2002), single imputation will be used in cases where the amount of missing data is low (< 1% of all data points). In all other instances, multiple imputation procedures will be applied to eliminate missing data in this study. The number of imputations will be 10.

DUHS IRB Application (Version 1.3)

General Information

***Please enter the full title of your protocol:**

Prospective Evaluation of Infant Development

***Please enter the Short Title you would like to use to reference the study:**

Prospective Evaluation of Infant Development
* This field allows you to enter an abbreviated version of the Study Title to quickly identify this study.

Add Study Organization(s):

List Study Organizations associated with this protocol:

Primary Dept?	Department Name
◆	**DUHS** - Duke Default Department

Assign key study personnel (KSP) access to the protocol

*** Please add a Principal Investigator for the study:**

(Note: Before this study application can be submitted, the PI MUST have completed CITI training)

Dodge, Kenneth

3.1 **If applicable, please select the Key Study personnel: (Note: Before this study application can be submitted, all Key Personnel MUST have completed CITI training)**
*** Denotes roles that are not recognized in OnCore. Please select an appropriate role that is recognized in all clinical research applications (iRIS, OnCore, eREG, etc.)**

A) Additional Investigators, Primary Study Coordinator (CRC), and the Primary Regulatory Coordinator (PRC):

Goodman, William
Primary Regulatory Coordinator

B) All Other Key Personnel

Andrade, Laura
Interviewer/Surveyor
Bai, Yu
Statistician
Campbell, Anna

Collaborator*
Carrig, Madeline
Statistician
Edwards, Matthew
Data Manager
Martin, Melissa
Study Coordinator (Duplicate)*
Murphy, Robert
Sub-Investigator
Nousak, Philip
Data Manager
Quinn, Jeffrey
Collaborator*
Reeves, Krysta
Collaborator*
Rehder, Peter, Ph.D.
Collaborator (Duplicate)*
Skandar, Alexa
Interviewer/Surveyor

***Please add a Study Contact:**

Dodge, Kenneth
Goodman, William
Martin, Melissa

The Study Contact(s) will receive all important system notifications along with the Principal Investigator. (e.g., The study contact(s) are typically the Principal Investigator, Study Coordinator, and Regulatory Coordinator.)

Oncore

Please select the Library for your Protocol:

This field is used in OnCore. Determines the Reference Lists, Forms, Protocol Annotations, Notifications, and Signoffs available for the protocol. Protocols that require reporting to the NCI (National Cancer Institute), must select the Oncology library.

- ○ Oncology
- ◉ Non-Oncology

Protocol Application Type

Select the type of protocol you are creating:

Please see additional criteria and information in the policy titled "Reliance on the IRB of Another Institution, Organization, or an Independent IRB" on the **IRB web site**.

- ◉ Regular Study Application - Most common. The IRB will determine if the study is eligible for expedited review or requires full board review upon submission.
- ○ Application for Exemption from IRB Review - Includes Exempt, Not Human Subject Research, & Not Research.
- ○ External IRB Application - Any study using an external IRB as the IRB-of-Record.
- ○ Trainee Research While Away from Duke - Research conducted by medical students overseen by the Office of Curriculum & other student/trainee research away from Duke.
- ○ Individual Patient Expanded Access, Including Emergency Use - Use of an investigational product under expanded access, including emergency use of an investigational drug or biologic or emergency

use of an unapproved device.

Conflict of Interest

Do any of the participating study investigators or other key personnel (or their immediate family/significant other) have a financial or intellectual interest in, or are receiving compensation from, the sponsor or the drugs, devices or technologies used in this research?

○ Yes ◉ No

Are any key personnel an inventor of any of the drugs, devices or technologies used in this research?

○ Yes ◉ No

Do any key personnel have or anticipate (within the year) any financial relationships (e.g., consulting, speaking, advisory boards, patents, equity, options) that could be perceived to overlap or present a conflict of interest with the current research?

○ Yes ◉ No

Do any key personnel have a conflict of interest management plan (issued by the Duke University School of Medicine Research Integrity Office) with this company?

○ Yes ◉ No

Oversight Organization Selection

CRU (Clinical Research Unit) or Oversight Organization Selection:

Please select the CRU.

Campus Oversight Organization

The Clinical Research Unit that takes responsibility for this study.

- More information on CRUs can be found on the Duke Office of Clinical Research (DOCR) website, **http://docr.som.duke.edu**
- Questions concerning CRU selection should be directed to docr.help@dm.duke.edu.
- For questions about the Campus Oversight Organization, please visit **Campus Oversight Organization**.

List all Key Personnel on the study who are outside Duke:

- **Note:** You will also need to attach the documentation of Human Subjects Certification for each individual, if they have completed the certification somewhere other than Duke.
- **If outside key personnel will have access to Duke PHI, a data transfer agreement AND external site IRB approval (or IRB authorization agreement) will be needed.** See HRPP policy **Use of Research Data by Former Duke Students or Former Duke Faculty and Employees**
- In the panel below, "PHI" is Protected Health Information.

Entry 1

**Name**	
**Study Role**	
**Email Address**	
**Institution / Organization**	
**Will he/she have access to Duke P.H.I.?**	○ Yes ○ No
**Is he/she an unpaid volunteer at Duke on the study?**	○ Yes ○ No

Indicate the Protocol source below:

The protocol source is the author of the protocol. If the protocol is a joint authorship between multiple sources, select the primary author.

An IRB fee may be assessed for all research that is supported by for-profit entities and requires full board review. For additional information, see the **IRB fees section of the IRB web site**

- (●) PI initiated
- ○ Commercial / Industry (for-profit entity) initiated
- ○ Federal Government initiated
- ○ Cooperative Group Initiated
- ○ Foundation (non-profit group) initiated
- ○ Other

Sponsor and Funding Source

Add all funding sources for this study:

View Details	Sponsor Name	Sponsor Type	Contract Type:	Project Number	Award Number
⊟	Duke Endowment	Institutional	Grant		

Sponsor Name:	Duke Endowment
Sponsor Type:	Institutional
Sponsor Role:	Funding
**Grant/Contract Number:**	16-06-SGO
**Project Period:**	From:01/01/2007 to:12/31/2018
**Is Institution the Primary Grant Holder:**	Yes
Contract Type:	Grant
Project Number:	
Award Number:	
Grant Title:	Prevent Child Abuse and Neglect Initiative
PI Name: (If PI is not the same as identified on the study.)	Kenneth Dodge
Explain Any Significant Discrepancy:	

View Details	Sponsor Name	Sponsor Type	Contract Type:	Project Number	Award Number
⊟	National Institute of Child Health and Human	Federal Government			

Development

Sponsor Name:	National Institute of Child Health and Human Development
Sponsor Type:	Federal Government
Sponsor Role:	Funding
**Grant/Contract Number:**	5R01 HD069981
**Project Period:**	From:04/01/2012 to:07/31/2023
**Is Institution the Primary Grant Holder:**	Yes
Contract Type:	
Project Number:	
Award Number:	
Grant Title:	Community Prevention of Child Maltreatment
PI Name:<br>(If PI is not the same as identified on the study.)	Kenneth Dodge
Explain Any Significant Discrepancy:	

Is this a federally funded study?

(•) Yes ( ) No

Does this study have any of the following?

- Industry sponsored protocol
- Industry funded Duke protocol
- Industry funded sub-contract from another institution
- Industry provided drug/device/biologic
- SBIR/STTR funded protocol

( ) Yes (•) No

As part of this study, will any samples or PHI be transferred to/from Duke to/from anyone other than the Sponsor, a Sponsor subcontractor, or a Funding Source?

( ) Yes (•) No

Is the Department of Defense (DOD) a funding source?

( ) Yes (•) No

For Federally funded studies:

Is your funding subject to, and does it comply with, the funding agency's policy for data sharing?

( ) Yes (•) No

Enter the Grant Number or Other Federal Agency Proposal or Application Number:

5R01 HD069981

Note: The Federal Funding Agency ID Number is the Sponsor's grant number assigned to your project and available on your Notice of Award (example: R01HL012345).

If known, enter the SPS (Sponsored Projects System) number if applicable:

2031814

In the Initial Submission Packet, attach the following:
(1) The entire grant, or an explanation of why a grant is not needed.
(2) NIH institutional Certificate form related to data sharing (if applicable).

The entire grant is needed so that it may be reviewed against the protocol for concordance.

Have you successfully synced your protocol to OnCore by clicking the 'Sync Data Over API' button at the top of this page?

Please verify that the protocol has been created in OnCore before submitting this application for PI Signoff.

- (•) Yes, I synced my protocol to OnCore and verified it was successfully sent by logging into OnCore.
- ( ) I may have forgotten! I'll click it again right now, just to be sure, and verify it was successfully sent by logging into OnCore.

Mobile Devices and Software

Does this study involve the use of a software or a mobile application?

( ) Yes (•) No

List all software, including third party (non-Duke) and mobile apps, that will be utilized for ascertainment, recruitment, or conduct of the research/project: (eg, MaestroCare, DEDUCE):

Multi-site Research

Is this a multi-site study?

( ) Yes (•) No

Complete for each site if Duke is the Primary grant awardee or coordinating center:

Entry 1

**Site Name:**	
**City:**	
**State/Province:**	
**Country:**	

Site Contact Information

**Primary Contact Name:**	
**Primary Contact Phone:**	
**Primary Contact Email:**	

Site Details

**Does the site have an IRB?**	○ Yes ○ No
**Site IRB approval expiration date:**	
**If date not provided, explanation of why:**	
**Has the site granted permission for the research to be conducted?**	○ Yes ○ No
**Does the site plan to rely on the DUHS IRB for review?**	○ Yes ○ No
**What is the status of the study at this site?**	○ Open<br>○ Closed
**Site approval letters or site personnel lists:**	Attach site approval letters, site closure letterS (if applicable), or site personnel lists in the Initial Submission Packet.

Research Abstract

Please type your Research Abstract here:

The Research Abstract should summarize the main points of your study in one paragraph. The following guidelines may help you:

1. Purpose and objective (1-2 sentences)
2. Study activities and population group (2-4 sentences)
3. Data analysis and risk/safety issues (1-2 sentences)

The purpose of the proposed study is to evaluate the effects on early child development of early community services, including a new, community-wide nurse home visiting program, "Durham Connects." Outcomes include: 1) child maltreatment; 2) child health and development and parenting; and 2) family service receipt and children's connection to a medical home. Participants (N=565) will be randomly selected and recruited from publicly available county birth records (one participant will be randomly selected for each birth date). A research assessment will occur when infants are approximately 6 months of age, including a maternal interview about parenting, child health, receipt of medical services and identification of child's primary care medical home, family service receipt, and maternal self-efficacy, as well as staff observations of mother-infant interactions. Mothers will also be asked for permission to access pediatric, hospital/emergency department, DSS, birth, and Durham Connects program records, which will be reviewed for child health and family service needs and receipt, as well as evidence of child maltreatment until the child is 5 years of age. We anticipate that about half of the participants will have participated in the Durham Connects Program and about a quarter will have participated in other family community services. Analyses will examine child and family outcomes and service receipt as a function of previous service

participation (e.g., Durham Connects, other services). In comparison to the anticipated benefits to participants and others, the risks in this study are considered minimal, including only some discomfort and/or self-consciousness for participants.

Research Summary

State your primary study objectives

The purpose of the proposed study is to evaluate the effects on early child development of early community services, including a new, community-wide nurse home visiting program, "Durham Connects." This program recruits mothers in the hospital at birth, provides up to three home visits, establishes connections between the family and primary health care or other community providers, and then follows up one month later to confirm these referrals. For its first 18 months *only*, due to limited funding, Durham Connects will be implemented to half the community (approximately 4,800 total births), with all even-day births being offered the program, and all odd-day births not being offered the program. The proposed study will assess the impact of Durham Connects and other early community services on: 1) child maltreatment; 2) child health and development and parenting; and 3) family service receipt and children's connection to a medical home.

State your secondary study objectives

Please select your research summary form:

Standard Research Summary Template

This is the regular (generic) research summary template which is required for all regular applications (unless your protocol fits under the other research summary templates in this category). Use of these instructions is helpful for ensuring that the research summary contains all necessary elements.

Standard Research Summary

Purpose of the Study

- Objectives & hypotheses to be tested

The proposed study will assess the impact of Durham Connects and other early community services on: 1) child maltreatment; 2) child health and development and parenting; and 3) family service receipt and children's connection to a medical home.

Background & Significance

- Should support the scientific aims of the research

Child maltreatment is an urgent public health problem. In the United States in 2006, 905,000 children (12 per 1,000) were identified as victims of abuse or neglect (US DHHS, 2008). Infants experience the highest victimization rates, at 23 per 1,000 infants under age 1 (CDC, 2008). These alarming rates actually underestimate the extent to which children experience abusive and neglectful parenting, as indicated by studies of parents' self-reported parenting practices (Straus, Hamby, Finkelhor, Moore, & Runyan, 1998; Theodore et al., 2005). Additionally, non-adherence to medical care is exceedingly common (roughly 50% in children with chronic disease) (Litt & Cuskey, 1980) which may reflect significant undetected medical neglect and place children at risk for severe health consequences. Likewise, nearly 25% of children do not receive the recommended number of well visits (Yu et al., 2002).

To date, the most effective services to prevent early maltreatment have taken the form of long-term, intensive home visiting for high-risk pregnant women or new mothers selected on the basis of demographic or psychosocial characteristics (e.g., Olds, 2006). Only some of these programs have proven efficacious, however. Often effects are greater in one subgroup than another. Furthermore, by targeting select groups, the programs may systematically exclude the majority of eventual child maltreatment cases (Dodge, 2009). Moreover, such intensive programs may be cost-prohibitive for many communities, even if they are cost-beneficial in the long run. Another proposed approach is to provide less intensive services *uni versally*. No such program has been implemented at scale and evaluated in a community-wide trial, however.

Design & Procedures

- Describe the study, providing detail regarding the study intervention (drug, device, physical procedures, manipulation of the subject or the subject's environment, etc.). Discuss justifications for placebo control, discontinuation or delay of standard therapies, and washout periods if applicable. Identify procedures, tests and interventions performed exclusively for research purposes or more frequently than standard of care. Include alternative therapies, concurrent therapies discontinued per protocol, risk benefit ratio, and use of tissue/specimens. Discuss monitoring during washout periods if applicable. Include brief description of follow-up, if any.

Participants will be drawn randomly from publicly available birth records at a rate of one per birthdate. Research assessments will take place in family homes when infants are 6 months old. Procedures will include an introduction to the study, informed consent procedures, and a comprehensive demographic and psychosocial interview. The interview (approximately 1.5 to 2 hours long) will consist of a maternal interview about parenting, child health, receipt of medical services and identification of child's primary care medical home, family service receipt, and maternal self-efficacy, as well as staff observations of mother-infant interaction. For monolingual Spanish mothers and mothers who prefer Spanish language interviews, procedures will be conducted in Spanish. Research interviewers will not be provided with the infant's date of birth, and will not ask for the date of birth during the interview, in order not to introduce bias in data collection due to knowledge of program or control group membership. The interview responses will be entered directly into a laptop computer.

Child health assessments will be conducted through a medical chart audit following written parental consent. Consent for release of this information from medical providers will be obtained during the initial interview with the mothers. Chart audit will occur in the pediatric and/or family practice offices. Each child' s chart will be reviewed for health outcomes and adherence to medical care. The audit form will be directly entered into a laptop computer at the pediatric or family practice office.

All mothers will also be asked for consent to access other community service records in which the family might have participated, including Durham Connects records, to confirm any participation. We anticipate that about half of the participants will have participated in the Durham Connects program and about a quarter will have participated in other family community services. Analyses will examine child and family outcomes and service receipt as a function of previous service participation (e.g., Durham Connects, other services).

Additionally, following written parental consent, we will review and analyze records from the North Carolina Division of Social Services (DSS) and hospital and emergency department records from Duke and Durham/Duke Regional Hospital. Every 12 months, NC DSS records of alleged and substantiated child abuse and neglect are transferred to the Center for Child and Family Policy (Durham Family Initiative Part I: Archival Data Review, IRB #Pro00010890). Our staff will review these records to search (using a SAS matching program and a manual review of the data) for any indication that our research participants were alleged or substantiated victims of abuse or neglect or had maltreatment-related diagnoses up to age 5. Additionally, Duke University and Durham/Duke Regional hospital admission and emergency department records will be requested every 12 months via the Duke Health Technology Systems (DHTS) data request website. Data on child healthcare utilization, as well as healthcare costs will be examined through child age 5, including dates of admission and discharge, type of visit, hospital and physicians billing costs, and relevant family demographic information. Hospital admission and emergency department records will also be reviewed for ICD-9 diagnostic codes for children associated with possible maltreatment. Consent for release of this information will be obtained during the initial 6-month interview with the mothers.

Mothers will also be asked for permission to access long-form birth records from the North Carolina State Center for Health Statistics. Family service participation as a function of infant and family status at birth will be examined.

In September 2014, the Home Visiting Evidence of Effectiveness group at the U.S. Department of Health and Human Services (HomVEE; **http://homvee.acf.hhs.gov/**) conducted a review of the Durham Connects program and its evidence base, based on two recent publications of Durham Connects program impact resulting from this protocol:

Dodge, K.A., Goodman, W.B., Murphy, R.A., O'Donnell, K., Sato, J., & Guptill, S. (2014). Implementation and randomized controlled trial evaluation of universal postnatal nurse home visiting [Special Issue]. *American Journal of Public Health*, *104*, S136-S143.

Dodge, K.A., Goodman, W.B., Murphy, R.A., O'Donnell, K., & Sato, J. (2013). Randomized controlled trial evaluation of universal postnatal nurse home visiting: Impacts on child emergency medical care at age 12-months [Special Issue]. *Pediatrics*, *132*, S140-S146.

This review, undertaken without the knowledge or consent of our research team, concluded that our impact evaluation study design was "low quality", in part, because the evaluation study had not established baseline equivalence for the evaluation sample according to criteria established by HomVEE. Results from this review are scheduled to be published on the HomVEE Model Reports website: **http://homvee.acf.hhs.gov/programs.aspx**.

In order to respond to this program review from HomVEE, and to ensure baseline equivalence of the random subsample of families participating in this Durham Connects impact evaluation study, we propose to obtain and examine data from long-form birth records for all eligible families that gave birth during the Durham Connects RCT implementation period from July 1, 2009 – December 31, 2010 (approximately 4,800 families) to ensure that 1) families participating in this impact evaluation study (n=551) are representative of the full population of births during this period (n=4,777); and 2) that no meaningful differences are observed between even birthdate (treatment eligible) and odd birthdate (services as usual) families. Comparisons will be conducted across multiple individual and family characteristics at birth (i.e., pre-intervention), including mother education, WIC/Medicaid status, and marital status. Data would be obtained from the Vital Records office at the NC Department of Health and Human Services only after obtaining approval from both the Duke School of Medicine IRB and NC DHHS to undertake this work.

As noted above, written informed consent to examine long-form birth records was obtained for the 551 families participating in this impact evaluation study. Written consent, however, was not obtained for the entire population of 4,777 births. We are requesting exempt status or an informed consent waiver *for this portion of the protocol only*, for the following reasons:

1. These analyses will be conducted in response to an external review from the Federal Government – this is not an original research question proposed by the study investigators.

2. These analyses will not produce generalizable knowledge - these analyses will only be used to addressed concerns raised by HomVEE regarding whether 1) the random subsample of families participating in this study are representative of the full population of eligible births that occurred during the 18-month Durham Connects RCT implementation period; and 2) whether the even / odd birthdate randomization strategy used in this RCT resulted in any systematic bias in treatment /control group assignment.

3. Obtaining informed consent from all 4,777 families is not practical/possible at this point in the study.

4. All data analyses would be conducted in aggregate – no individual identifiers will be examined or reported.

Selection of Subjects

- List inclusion/exclusion criteria and how subjects will be identified.

We will use publicly available short-form birth records to identify eligible participants. The birth records include key information needed to contact eligible families, including parent names, child name, child date of birth, family address, parents' race/ethnicity, and hospital of birth.

The research team will randomly select one birth per day (July 1, 2009 – June 30, 2010) of every month (total: 18) for participation in the proposed study. Only infants born in Durham County hospitals will be recruited. A total of 565 participants will be enrolled. It is estimated that the initial random selection will result in a sample that is representative of the births in Durham County. To prepare for cases in which we are unable to reach the initially drawn subject, we will produce a 2:1 replacement list at the

time of initial drawing. Replacements will be the two births that occur closest in time to the originally drawn birth who match the originally drawn birth on even/odd birth date as well as on race/ethnicity (defined as White/Non-Hispanic, Black/Non-Hispanic, White/Hispanic, and other).

Subject Recruitment and Compensation

- Describe recruitment procedures, including who will introduce the study to potential subjects. Describe how you will ensure that subject selection is equitable and all relevant demographic groups have access to study participation (per 45 CFR 46.111(a) (3)). Include information about approximately how many DUHS subjects will be recruited. If subjects are to be compensated, provide specific prorated amounts to be provided for expenses such as travel and/or lost wages, and/or for inducement to participate.

Using the contact information in the publicly-available birth records, recruitment will be completed through phone calls, letters, and door-to-door recruiting. In order to best reach families, we will contract with a survey research company to back match available addresses with telephone numbers. It expected that we will attain a telephone retrieval rate between 40% and 50%. Additional recruiting strategies (letters, door-to-door recruiting) will be used to reach families without a listed phone number. In the event that information extracted from birth records is insufficient or inaccurate, we aim to use methods such as publicly available social networking sights, such as Facebook or MySpace, as well as White Pages, Autotrack, and other methods of searching for people on publicly available networks. On social networking sites, we will search for participants by their names. If multiple names manifest, we will look to see who is of an age that they could be a mother of a 6-month old and confirm that they live in the Durham area. Sometimes, their profile picture may in fact show the participant's baby or her profile may name the baby. This would further confirm our finding as accurate. Once we determine that a finding is a viable candidate for the target participant, we will send the potential participant a general message to verify that she has a 6-month old child. We will use our phone script as a template for sending an initial message to candidates found on these social networking sights, and ask the potential participant to call us to learn more about the study or to schedule an interview, once we have confirmed that they are the target participant. For participating in the interview, participants will be paid $50 and children will be given small, age-appropriate toys (valued at $5).

Consent Process

- Complete the consent section in the iRIS Submission Form.

Subject's Capacity to Give Legally Effective Consent

- If subjects who do not have the capacity to give legally effective consent are included, describe how diminished capacity will be assessed. Will a periodic reassessment occur? If so, when? Will the subject be consented if the decisional capacity improves?

Cognitive capacity will be assessed through interactions and the interview itself, and potential participants with known or suspected cognitive impairment will not be recruited. Mental health needs are a part of the existing interview, and potential participants who present with acute and emergent needs will not be eligible for inclusion. Any participating mothers under 18 years of age will be informed of the study with a custodial parent present, and consent will be requested from both the parent and the teenaged mother.

Study Interventions

- If not already presented in #4 above, describe study-related treatment or use of an investigational drug or biologic (with dosages), or device, or use of another form of intervention (i.e., either physical procedures or manipulation of the subject or the subject's environment) for research purposes.

N/A

Risk/Benefit Assessment

- Include a thorough description of how risks and discomforts will be minimized (per 45 CFR 46.111(a) (1 and 2)). Consider physical, psychological, legal, economic and social risks as applicable. If vulnerable populations are to be included (such as children, pregnant women, prisoners or cognitively impaired adults), what special precautions will be used to minimize risks to these subjects? Also identify what available alternatives the person has if he/she chooses not to participate in the study. Describe the possible benefits to the subject. What is the importance of the knowledge expected to result from the research?

This study is expected to pose minimal risks to participating mothers and their children. The proposed procedures and measures have been used widely in developmental psychology research involving participants with no identified problems as well as those with very pronounced problems. There are no physical risks to participants. Portions of the assessment measures may cause some temporary discomfort or uneasiness; however serious adverse effects are unlikely and have not been encountered in our work to date or, to our knowledge, in previous studies with these measures. As noted above, participants will be reminded that they are free to decline to answer any question, that they may stop answering or withdraw consent for sharing their administrative record data at any time without penalty, and that steps will be taken to ensure the confidentiality of their data. There is some possibility that participants may experience discomfort in answering interview questions and that the interview may identify emergent clinical needs (e.g., depression, substance use problems). If a participant's responses indicate that referrals are appropriate (e.g., participant indicates distress that is impairing their functioning, participant expresses interest in seeking clinical help), research staff will provide information on referrals for appropriate counseling and/or substance use treatment services.

One risk for participants is the potential loss of confidentiality when child maltreatment records are reviewed. Great care will be taken to ensure that confidentiality is protected for all participants, as described in section 13. Additionally, it is possible that observations and maternal reports to research assistants may reveal information regarding harm or serious risk of harm to children which would be subject to mandatory reporting requirements. Prior to each assessment session, participants will be informed about mandatory reporting and the risks of disclosure, and that they may choose to skip the items that cause them to feel uncomfortable. In an effort to minimize risk associated with reporting child maltreatment, research staff will discuss the issue with mothers prior to making any report, except when it is deemed dangerous to the child or to the person filing the report to do so. We will permit mothers to be involved in reporting the maltreatment themselves if they choose, which can reduce the stress that a mother may experience as a result of a report being made.

Information from the study may directly benefit the women in the study by giving them the opportunity to reflect on their family's circumstances and experiences. In the longer run, findings are expected to advance the field of child maltreatment prevention through the evaluation of community-wide programs that aim to deliver effective, personalized care.

In comparison to the anticipated benefits to participants and others, most of the risks to participants in this study are minimal, including only some discomfort and/or increased self-consciousness for mothers. More serious risks to participants, such as being reported for child maltreatment, are expected to occur infrequently. In sum, the minimal risks to participants are reasonable in relation to the importance of the knowledge that may reasonably be expected to result from the proposed study.

Costs to the Subject

- Describe and justify any costs that the subject will incur as a result of participation; ordinarily, subjects should not be expected to pay for research without receiving direct benefit.

Study participants will not incur any costs as a result of participation.

Data Analysis & Statistical Considerations

- Describe endpoints and power calculations. Provide a detailed description of how study data will be analyzed, including statistical methods used, and how ineligible subjects will be handled and which subjects will be included for analysis. Include planned sample size justification. Provide estimated time to target accrual and accrual rate. Describe interim analysis including plans to stop accrual during

monitoring. Phase I studies, include dose escalation schema and criteria for dose escalation with definition of MTD and DLT.

Statistical analyses involved in this project include basic descriptive analyses and examination of child and family outcomes and service receipt as a function of previous service participation (e.g., Durham Connects, other services). The primary research question is whether the service participation results in better child and family outcomes (i.e., fewer official reports of maltreatment, more enhanced parent report of parenting and child health and development outcomes) than does no service participation. The second research question will examine if Durham Connects program families receive more enhanced family services and are connected to a medical home (i.e., by maternal report and verification from medical records, there is a regular medical provider/primary care physician for mother and infant, infant attends well-child check ups, infant does not attend emergency department for routine care), and whether program mothers report higher self-efficacy. We will conduct all analyses using an intent-to-treat approach. Though concerted efforts will be made to maintain the maximum sample possible, some missing observations are expected. Full information maximum likelihood (FIML) estimation will be applied to the multivariate regression models to handle missing data, in order to reduce bias in the parameter estimates and to preserve statistical power.

DSS reports are likely to occur at relatively low frequency, particularly given that reporting may greatly underestimate actual maltreatment rates. To maximize power, survival analyses will be used to examine group differences. Child and family outcomes with continuous scores and greater variability will also be examined. Using power calculation software, we found the study to be adequately powered to detect significant differences for our principal hypotheses.

Data & Safety Monitoring

- Summarize safety concerns, and describe the methods to monitor research subjects and their data to ensure their safety, including who will monitor the data, and the frequency of such monitoring. If a data monitoring committee will be used, describe its operation, including stopping rules and frequency of review, and if it is independent of the sponsor (per 45 CFR 46.111(a) (6)).

Data security will be carefully monitored in the data collection, processing, and analysis stages. The data will be stored on a secure network server, with password protection such that only authorized users will have access to the file server. Original data will be kept in locked compartments separate from documentation and access information. Any temporary data files kept on removable storage devices, as well as printouts derived from data analysis, will be stored in a locked compartment when not in use. All electronic and paper files that contain identifying information will be destroyed at the conclusion of the research. Published data from the proposed study will be in the form of group-level data and will not permit identification of individuals.

Privacy, Data Storage & Confidentiality

- Complete the Privacy and Confidentiality section of the iRIS submission form.

Describe Role of External Personnel:

N/A

Study Scope

Does the subject population contain >50% malignant hematology or oncology patients, or their caregivers?

○ Yes ◉ No

Are you using a drug, biologic, food, or dietary supplement in this study?

( ) Yes (•) No

Are you using a medical device, an algorithm (whether computer based or not), an in vitro diagnostic test, or using samples to look for biomarkers in this study?

( ) Yes (•) No

Does this study employ magnetic resonance, including imaging (MRI), spectroscopy (MRS), angiography (MRA) or elastography (MRE) beyond the standard of care?

( ) Yes (•) No

Does this study specify or require the performance of diagnostic procedures using ionizing radiation (x-rays, DEXA, CT scans, nuclear medicine scans, etc.) that are beyond the standard of care?

( ) Yes (•) No

Does this study specify or require the performance of therapeutic procedures using ionizing radiation (accelerator, brachytherapy or systemic radionuclide therapy) that are beyond the standard of care?

( ) Yes (•) No

Will the participant be subjected to increased or decreased ambient pressure?

( ) Yes (•) No

Do you plan to recruit subjects from Duke Regional Hospital (DRH)?

( ) Yes (•) No

Do you plan to recruit subjects from Duke Raleigh Hospital (DRAH)?

( ) Yes (•) No

Does this study utilize the Duke Early Phase Clinical Research Unit (DEPCRU)?

( ) Yes (•) No

Are you using the Duke logo in any advertisements?

( ) Yes (•) No

Is this study retrospective, prospective, or both?

"Retrospective" means that data or samples already in existence (collected prior to the study submission) will be used.
"Prospective" means there will be data or samples collected in this study for research purposes.

( ) Retrospective
(•) Prospective
( ) Retrospective and Prospective

If the study is both retrospective and prospective: Is this a review soley of information collected for non-research purposes (i.e. a review of medical records)?

○ Yes ○ No

Does this protocol include any research using botulinum toxin, including the FDA-approved clinical product (Botox)?

○ Yes ◉ No

Does this protocol involve the administration of any of the following materials to humans?

•Any viral vector or plasmid
•Any cells that have been modified by a viral vector
•Any other genetically-modified cells
•Any genetically-modified virus, bacterium, or other agent
•Any other recombinant or synthetic nucleic acid

○ Yes ◉ No

Subject Population Groups and Enrollment

Population Groups (Select targeted population groups only):

Note:

- If Minors are included, the study will be routed to the Department of Pediatrics for Pediatric Risk Assessment.
- Students and Employees over whom Key Personnel have a supervisory role may not be enrolled in this study

- [x] Adults
- [ ] Minors who are Wards of State
- [x] Minors
- [ ] Duke Patients
- [ ] Pregnant Women
- [ ] Fetuses
- [ ] Prisoners
- [ ] Adults incapable of giving consent
- [ ] Adults with diminished capacity
- [ ] Handicapped subjects
- [ ] Students
- [ ] Employees
- [ ] Healthy Controls
- [ ] Deceased subjects
- [ ] Blanket Protocol

This study will be routed to the Department of Pediatrics for Pediatric Risk Assessment.

Please select any population groups excluded from participation in this study:

- [ ] Pregnant women

Maximum number of subjects to be consented at Duke:

Enter a single number. If you anticipate consenting a range of subjects, enter the **upper** limit of the range. The number should represent the maximum number of subjects for the life of the study.

565

Maximum number of subjects to be consented at all sites:

Enter a single number. If you anticipate consenting a range of subjects, enter the **upper** limit of the range. The number should represent the maximum number of subjects for the life of the study.

565

Subject Procedures and Costs

Biobank - Does this study involve the collection, use, tracking, banking (storage) or distribution of human biological specimens?

Human biological specimens include blood or its components, healthy or diseased tissue, bodily fluids, DNA /RNA or human stem cells.

○ Yes ◉ No

Procedures

Check all the apply:

- ☐ Genetic Testing
- ☐ Gene Transfer
- ☐ DNA Banking
- ☐ Testing for Reportable Infectious Diseases
- ☐ Human Cell Banking
- ☐ *Use of Human Embryonic Stem Cells
- ☐ *Use of Human-induced Pluripotent Stem Cells
- ☐ *Use of Other Cells Derived from Human Embryos
- ☐ *Use of Human/Animal Chimeric Cells
- ☐ *Specialized Cell Populations for Cell Therapy
- ☐ Use of Human Tissue
- ☐ Use of Bodily Fluids
- ☐ Use of Blood (or its components)
- ☑ Not Applicable

Will blood be drawn in this study for research purposes?

○ Yes ◉ No

Will the Operating Room be used in this study?

Include only research time, not clinical care time.

○ Yes ◉ No

Will there be extra costs to subjects or insurance as a result of the research (e.g. tests, hospitalization)?

○ Yes ◉ No

Will there be Subject Compensation?

(●) Yes ( ) No

Compensation for Travel / Lost Income (in USD):

50

Other Subject Compensation:

Toy for participant's child, valued at $5

Subject Recruitment Materials

For each document to be reviewed, use the table below to provide the following information:

Attach a copy of each advertisement that you will be using with this study in the Initial Submission Packet. If any Ad will have multiple wording variations, attach a copy of each version of the Ad.
All materials that will be used to advertise the study in order to recruit subjects must be approved by the IRB.

Types of subject recruitment materials include, but are not limited to, the following:

Direct Advertising
Posters
Billboards
Flyers
Brochures

Media Advertising
Newspaper Ads
Magazine Ads
Radio Ads
TV commericals / Video
Internet website
Social Media

Other Types of Advertising
Newsletter
Email
Postcards / Letters

(Note: Doctor-to-Doctor letters do not require IRB approval)

Document name	Material category	Location material displayed	Has this material previously been approved by the IRB?
Recruitment Phone Script - English	( ) Billboard / Flyer / Poster<br>( ) Brochure<br>( ) Internet website / Email<br>( ) Letter / Postcard<br>(●) Phonescript<br>( ) Radio<br>( ) Television / Video<br>( ) Newsletter / Newspaper /	Please be specific. For example, "Duke" would not be an appropriate location. "Duke Hospital Television" would be an appropriate response.<br><br>English language phone script read	( ) Yes (●) No

	○ Magazine<br>○ Other	during call with potential study participants	
Recruitment Letter - English	○ Billboard / Flyer / Poster<br>○ Brochure<br>○ Internet website / Email<br>◉ Letter / Postcard<br>○ Phonescript<br>○ Radio<br>○ Television / Video<br>○ Newsletter / Newspaper / Magazine<br>○ Other	Please be specific. For example, "Duke" would not be an appropriate location. "Duke Hospital Television" would be an appropriate response.<br>English language letter mailed to potential study participants	○ Yes ◉ No
Recruitment Phone Script - Spanish	○ Billboard / Flyer / Poster<br>○ Brochure<br>○ Internet website / Email<br>○ Letter / Postcard<br>◉ Phonescript<br>○ Radio<br>○ Television / Video<br>○ Newsletter / Newspaper / Magazine<br>○ Other	Please be specific. For example, "Duke" would not be an appropriate location. "Duke Hospital Television" would be an appropriate response.<br>Spanish language phone script read during call with potential study participants	○ Yes ◉ No
Recruitment Letter - Spanish	○ Billboard / Flyer / Poster<br>○ Brochure<br>○ Internet website / Email<br>◉ Letter / Postcard<br>○ Phonescript<br>○ Radio<br>○ Television / Video<br>○ Newsletter / Newspaper / Magazine<br>○ Other	Please be specific. For example, "Duke" would not be an appropriate location. "Duke Hospital Television" would be an appropriate response.<br>Spanish language letter mailed to potential study participants	○ Yes ◉ No
Recruitment Phone Script - Spanish Back Translation	○ Billboard / Flyer / Poster<br>○ Brochure<br>○ Internet website / Email<br>○ Letter / Postcard<br>◉ Phonescript<br>○ Radio<br>○ Television / Video<br>○ Newsletter / Newspaper /	Please be specific. For example, "Duke" would not be an appropriate location. "Duke Hospital Television" would be an appropriate response.<br>English language back translation of	○ Yes ◉ No

	Magazine<br>○ Other	Spanish language phone script	
Recruitment letter - Spanish language back translation	○ Billboard / Flyer / Poster<br>○ Brochure<br>○ Internet website / Email<br>◉ Letter / Postcard<br>○ Phonescript<br>○ Radio<br>○ Television / Video<br>○ Newsletter / Newspaper / Magazine<br>○ Other	Please be specific. For example, "Duke" would not be an appropriate location. "Duke Hospital Television" would be an appropriate response.<br>English language back translation of Spanish language letter mailed to potential study participants	○ Yes ◉ No

Consent Process

Attach draft consent forms in the Initial Review Submission Packet.

Consent forms must be MS Word documents and follow the specific format outlined by the IRB. **Click here** to download a copy of the consent form template.

Note: Please do not edit the section of the footer that contains the Protocol ID, Continuing Review and Reference Date fields. Those fields will be used to stamp the final consent form when it is approved by the IRB. If you want to add an internal version date, please put it in the header.

Who will conduct the consent process with prospective participants?

Give the person's role in this study (PI, Study Coordinator, etc.):

The consent process will be conducted by the interviewers for the project. All interviewers will have completed Duke IRB ethics certification and will be trained in consenting procedures.

Who will provide consent or permission?

(Select all that apply):

- [x] Participant
- [x] Parent(s) or Legal Guardian(s)
- [ ] Legally Authorized Representative (LAR)

How much time will the prospective participant (or legally authorized representative) have between being approached about participating in the study and needing to decide whether or not to participate?

If you are not giving the person overnight to consider whether or not to participate, please justify.

Participants will have between the time they are recruited by phone or in person to the time of the interview. We anticipate that this span of time will be no less than one day, and generally several days to a week in advance.

Where will the consent process occur?

The consent process will occur in participants' homes.

What steps will be taken in that location to protect the privacy of the prospective participant?

Every effort will be made to protect the privacy of individuals during the consent process and research interview. Participants will be informed in advance (during recruitment contact) about the length and nature of the interview, and will suggest having a quiet, private space to complete the interview. During the interview in the home, if other individuals are present or within hearing distance, the interviewer will assess the participants' comfort level with others' presence, and inquire about alternative locations or solutions.

How much time will be allocated for conducting the initial consent discussion, including presenting the information in the consent document and answering questions, with each prospective participant?

Based on our research with similar populations, we anticipate that the consent discussion (presentation and answering questions) will take approximately 10-20 minutes, depending on the needs and questions of the participants. No set time will be determined, and the time needed by the participant will be allotted.

What arrangements will be in place for answering participant questions before and after the consent is signed?

Participants will be informed throughout the consent and interview process that they may ask questions at any time, and may take breaks if needed. Additionally, a copy of the signed consent form with contact information for the project staff will be left for the participant in case further questions arise.

Describe the steps taken to minimize the possibility of coercion or undue influence.

Efforts will be in place to minimize the possibility of coercion or undue influence. The interviewers obtaining consent in these interviews have no role of authority over the families (e. g., not a doctor or otherwise involved in the family's health or service needs). No services will be altered or denied based on any participant's lack of consent. Interviewers will explicitly make clear the fact that participation (in the interview as well as in answering any question within the interview) is completely voluntary and will not affect the family's medical or social services. A one-time payment of a $50 pre-paid Visa card along with a small toy for the child (valued at $5) will be provided to families. This amount is consistent with amounts we have provided in other studies and has been viewed as an appropriate token of appreciation for families' time.

What provisions will be in place to obtain consent from participants who do not read, are blind or who do not read/understand English?

Regarding participants who do not read or are blind, interviewers will offer to read the consent information aloud to the participants. Regarding non-English speakers, based on our work in the Durham community, we expect about 30% of the participants to be Spanish speaking. We will have Spanish-speaking interview staff conduct these interviews. All consent documents will be translated into Spanish and back-translated into English to ensure clear and appropriate translation. For speakers of other languages, we would need to contract with an official translator in that language, although based on our research and service work in this community we expect this to occur rarely.

Do you plan to obtain written consent for the conduct of research?

◉ Yes ○ No

Protected Health Information (PHI)

Indicate how you intend to use potential subjects' Protected Health Information (PHI):

○ I will review, but not record, PHI prior to consent.
○ I will record PHI prior to consent.
◉ I do not intend to use PHI prior to consent.
○ I will record PHI without consent. (decedent research, database repository, chart review)

Privacy and Confidentiality

Explain how you will ensure that the subject's privacy will be protected:

Consider privacy interests regarding time and place where subjects provide information, the nature of the information they provide, and the type of experience they will be asked to participate in during the research.

Considerable care will be given to avoiding inadvertent disclosure of confidential information about the study participants. Participant names will not be used on any paper records or in the interview database; they will be assigned confidential ID numbers. There will be a hard copy of a master list linking respondents, ID numbers, addresses, and phone numbers; this list and project consent forms (which contain identifying data) will be kept in a locked cabinet within the project offices to assure confidentiality. Staff members who are directly involved in the recruitment and data processing will have access to the identified data regarding the research interviews. All research staff will be required to sign a pledge of confidentiality that acknowledges each project member's responsibility to guard against unauthorized use or disclosure of any identifiable information about the participants. Interview data will be collected via laptops and secured, as described below.

In addition to the interview data, the Center for Child and Family Policy will also be the recipients of outside databases such as DSS and hospital records. As noted above, our center has already been granted permission to access and analyze DSS records between 1997 and 2012 by the North Carolina and Durham County Departments of Social Services and by the DUMC IRB. An electronic copy of the identified data will be accessible to a single data manager and the manager's assistant, both well-trained in confidentiality procedures and ethics. These individuals will be responsible for reviewing administrative data and matching up cases with identifying information for all those who have consented to have their DSS and hospital records followed. Once the data are matched and appropriately linked to the current study data, identifiers other than the study number will be removed, and the data will be shared with the statistician and investigators for analysis via a file on a secure server. The database with DSS and hospital data attached will remain in separate files on the secure server, accessible only to the data manager and the assistant. Identified data will thus never be viewed by other study staff (e.g., statisticians or investigators).

Describe how research data will be stored and secured to ensure confidentiality:

How will the research records and data be protected against inappropriate use or disclosure, or malicious or accidental loss or destruction? Records and data include, for example, informed consent documents, case report forms or study flow sheets, survey instruments, database or spreadsheets, screening logs or telephone eligibility sheets, web based information gathering tools, audio/video/photo recordings of subjects, labeled specimens, data about subjects, and subject identifiers such as social security number.

All project data will be stored in locked cabinets in secured project offices. Electronic data gathered in home visits and pediatric offices will be on password-protected and encrypted on laptops. Padlocks will be secured on the laptop carry cases when the laptops are not in use. All data will be removed from the laptops within 24 hours and uploaded and backed up to a secure network server with password protection.

Application Questions Complete

Please click Save & Continue to proceed to the Initial Submission Packet.

The Initial Submission Packet is a short form filled out after the protocol application has been completed. This is an area to attach protocol-related documents, consent forms, and review the application.
