# Supplementary material for: Effect of a Universal Postpartum Nurse Home Visiting Program on Child Maltreatment and Emergency Medical Care at 5 Years of Age: A Randomized Clinical Trial
Source: JAMA Netw Open. 2021 Jul 7;4(7):e2116024. doi: 10.1001/jamanetworkopen.2021.16024 (PMC8264647; doi:10.1001/jamanetworkopen.2021.16024)
Supplement: Supplement 2. — Data Sharing Statement [file jamanetwopen-e2116024-s002.pdf]

Data Sharing Statement

Goodman. Effect of a Universal Postpartum Nurse Home Visiting Program on Child Maltreatment and Emergency Medical Care at 5 Years of Age. *JAMA Netw Open*. Published July 07, 2021. doi:10.1001/jamanetworkopen.2021.16024

Data
Data available: Yes
Data types: Deidentified participant data, Other (please specify)
Additional Information: Codebooks
How to access data: Requests should be sent to ben.goodman@duke.edu
When available: With publication

Supporting Documents
Document types: None

Additional Information
Who can access the data: Researchers with an IRB approval and approved proposal.
Types of analyses: Data will be made available for analyses proposed in the approved study proposal.
Mechanisms of data availability: Sensitive Box folder (approved by the Duke School of Medicine IRB for storage of HIPAA and ePHI data)
Any additional restrictions: None
